# Aging and Thermoregulatory Control: The Clinical Implications of Exercising under Heat Stress in Older Individuals

**DOI:** 10.1155/2018/8306154

**Published:** 2018-08-02

**Authors:** Bryce N. Balmain, Surendran Sabapathy, Menaka Louis, Norman R. Morris

**Affiliations:** ^1^School of Allied Health Sciences, Griffith University, Gold Coast, Australia; ^2^Metro North Hospital and Health Service, Allied Health Research Collaborative, The Prince Charles Hospital, Brisbane, Australia

## Abstract

Climate change is predicted to bring about a greater variability in weather patterns with an increase in extreme weather events such as sustained heat waves. This change may have a direct impact on population health since heat waves can exceed the physiological limit of compensability of vulnerable individuals. Indeed, many clinical reports suggest that individuals over the age of 60 years are consistently the most vulnerable, experiencing significantly greater adverse heat-related health outcomes than any other age cohort during environmental heat exposure. There is now evidence that aging is associated with an attenuated physiological ability to dissipate heat and that the risk of heat-related illness in these individuals is elevated, particularly when performing physical activity in the heat. The purpose of this review is to discuss mechanisms of thermoregulatory control and the factors that may increase the risk of heat-related illness in older individuals. An understanding of the mechanisms responsible for impaired thermoregulation in this population is of particular importance, given the current and projected increase in frequency and intensity of heat waves, as well as the promotion of regular exercise as a means of improving health-related quality of life and morbidity and mortality. As such, the clinical implications of this work in this population will be discussed.

## 1. Introduction

In the context of climate change, global surface temperatures are rising and the frequency, duration, and intensity of heat waves are projected to increase in the coming decades [1]. In countries like Australia, heat waves have been identified as being responsible for more deaths than all other types of natural disasters combined [2]. Around the world, over the past 20 years heat waves have led to extremely high levels of excess morbidity and mortality [3–5]. A classic example, and perhaps the most severe, is the 2003 Western European heat wave where an estimated 13,700 people died in France as a result of the increase in temperature [6]. In light of these events, climate change clearly poses a global threat of considerable magnitude for human health, the incidence of which is projected to increase especially in vulnerable populations.

Elderly individuals, particularly those who are over the age of 60 years, are one of the most vulnerable populations during environmental heat exposure, experiencing significantly greater adverse heat-related health outcomes than any other age cohort [7, 8]. Indeed, there is now evidence showing that older individuals have impaired thermoregulatory control [9–12] and that the risk of heat-related illness in these individuals is elevated, particularly when performing physical activity in the heat [13, 14]. Although regular physical activity leads to a reduction in health-related morbidity and mortality, individuals who do not have regular access to temperature-controlled exercise training facilities (i.e., gymnasiums) and/or in-home air-conditioning are therefore more likely restricted to performing physical activity outside of formal exercise training programs, which can take place under a range of environmental conditions, including outdoors in a warm environment (particularly in the summer months). Whilst current guidelines provide a general overview of the risk of exertional heat-related illness for younger individuals and athletic populations [15, 16], there are no public health recommendations for performing physical activity in the heat specifically for older individuals. In this brief review, we describe the normal thermoregulatory mechanisms and how these may be altered in older individuals. Additionally, the clinical relevance of this work is discussed; this is particularly relevant given that climate change, coupled with an aging population, is exposing an increasingly greater number of individuals, including those with chronic heart and lung disease, to the risks of heat-related morbidity and mortality.

## 2. Thermoregulatory Control

**Figure 1:**
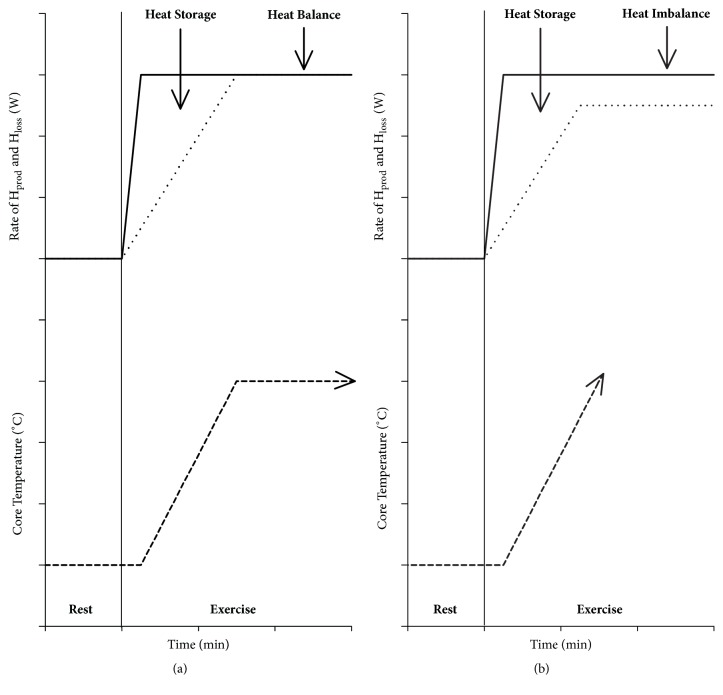
**(a) **At rest, the rate of H_prod_ (solid line) is balanced out by the rate of H_loss_ (dotted line), a combination of evaporative and dry heat exchange. At the onset of exercise, H_prod_ rapidly rises reaching a steady state within 5 min. Due to the delay in the onset of H_loss_ responses, H_loss_ does not immediately balance the rate of H_prod_ resulting in net heat storage. The additional heat energy stored inside the body initiates increases in sweating and skin blood flow to increase H_loss_ and achieve heat balance to prevent a continuing rise in core temperature (dashed line).** (b)** When heat balance cannot be achieved, core temperature will continue to rise to potentially dangerous limits and not plateau, due to limited H_loss_ capacity; the rate of H_loss_ required to achieve heat balance is greater than the maximum capacity for heat dissipation. H_prod_: metabolic heat production; H_loss_: net heat loss.

Humans maintain a relatively stable core body temperature of ~37°C despite being exposed to a wide variety of environmental conditions. Maintaining core body temperature within a safe, narrow range (i.e., between 35 and 39°C) ensures that metabolic reactions within the human body occur at a near-optimal level when possible [17]. When humans are exposed to heat stress (i.e., elevated environmental temperatures, physical activity, or a combination of both), the thermoregulatory system engages a number of physiological mechanisms to maintain heat balance. That is, the internal heat produced by cellular respiration (metabolic heat production [H_prod_]) is balanced out by the rate of heat that is lost from the skin surface to the surrounding environment through a combination of dry (conduction, convection, and radiation) and evaporative heat exchange (Figure 1, Panel (a)) [18, 19]. Increases in metabolic and muscular activity cause a rise in H_prod_ which is due to the inefficiency of the metabolic reactions required to provide energy to the working muscles [20]. Muscular contraction is extremely inefficient with approximately 80% of the energy generated produced as heat [21]. As such, the change in core body temperature secondary to the additional heat energy stored inside the body provides thermal afferent impulses to the central nervous system [22], which subsequently sends efferent signals to appropriate effector organs to initiate sustained increases in sweating and skin blood flow (SkBF) ensuring that core body temperature is maintained within safe limits.

In accordance with the fundamental heat balance theory [18], core body temperature will continue to rise if heat imbalance persists, whereby sweating and SkBF responses are not able to facilitate the required rate of heat loss during environmental and/or physical activity-induced heat stress (Figure 1, Panel (b)) [13]. Extended periods of heat stress can increase the likelihood of heat-related illness (particularly in older individuals) [13]. Major heat-related conditions, including heat stroke which is characterized as a severe elevation in body temperature that causes body tissue and central nervous system dysfunction [23, 24], all result from insufficient heat loss from the body.

In recent years, the changes in thermoregulatory control with aging have received some attention [13, 25–27]. These changes have been attributed to a combination of factors including alterations in sweating and SkBF (detailed below). Aside from these well-documented age-related changes, alterations in cardiovascular function with comorbid disease such as heart and lung disease can also contribute to altered thermoregulatory control in the older individual, particularly during exercise. Therefore, to maintain heat balance an optimally functioning thermoregulatory system is essential for humans to respond appropriately to environmental and/or physical activity-induced thermal challenges.

## 3. Age-Related Changes in Sweating

In humans, the thermoregulatory response with the greatest capacity for heat loss during environmental heat exposure and physical activity is the evaporation of sweat [29]. Under conditions permitting complete evaporation (i.e., when the maximal evaporative potential within a given environment does not limit an individual's ability to achieve heat balance), evaporation is the primary mechanism for heat loss [29, 30], with just one gram of sweat liberating 2426 J of heat energy upon evaporation [31]. To date, evidence suggests that older individuals exhibit alterations in sweating during heat stress compared to younger, gender-matched individuals [7, 11, 12, 27, 32–35]. A common finding amongst studies is that older individuals demonstrate a delayed core temperature onset threshold for sweating and a reduction in evaporative heat loss (due to a lower overall sweat rate) compared to their younger healthy counterparts. These age-related decrements in sweating do not appear to be due to a reduction in the number of activated sweat glands, but rather to a reduction in the amount of sweat produced per gland, as shown by studies in which sweat glands were pharmacologically stimulated [35, 36]. The decrease in sweat gland output with aging may reflect age-related changes in sweat glands themselves (sweat gland atrophy) or a decrease in cholinergic sensitivity [37]. Moreover, additional findings indicate that regional differences in sweat gland function exist between older and younger groups [38]. Indeed, greater age-related effects have been commonly reported for sweat gland function on the forehead and limbs when compared to sweat glands located on the trunk. In light of these findings, it has been suggested that sweat gland function may decline in a peripheral-to-central direction as skin ages [27].

With respect to examining sweating responses during exercise in the heat, Kenney and Anderson [39] demonstrated that local sweat rate was less in older individuals than their younger counterparts during exercise at a relative intensity (40% of maximal oxygen uptake) in the heat. Tankersley et al. [40] and Inbar et al. [14] demonstrated that local and whole-body sweat rate was lower in older individuals compared to younger individuals during exercise at 65% and 50% of maximal oxygen uptake, respectively, in a hot (30°C and 41°C, respectively) environment. The attenuated evaporative heat loss capacity found in older individuals results in greater heat energy stored inside the body which can cause core body temperature to rise to potentially dangerous levels. Whilst these findings [14, 39, 40] support the above evidence in that the capacity to produce sweat during heat stress decreases with advancing age, it must be noted that differences in biophysical properties (body mass and surface area, and absolute external work load) associated with H_prod_ were not controlled in these studies.

Recent studies have shown that biophysical differences can independently influence sweating responses, particularly during exercise [29, 41–44]. For example, an individual with a small body surface area would require a greater amount of sweat to be produced per unit body surface area to achieve the same absolute rate of evaporation compared with an individual with a larger body surface area. This is consistent with the findings of Cramer and Jay [41], who demonstrated that local sweat rate was significantly higher in individuals with a smaller body surface area (~1.8 m^2^) compared with individuals with a larger body surface area (~2.1 m^2^) during exercise at any given fixed rate of evaporative requirement and thus H_prod_. Hence, it is difficult to determine whether the aforementioned findings are due to aging* per se* [14, 39, 40] or can be purely ascribed to between-group biophysical differences associated with H_prod_. In this regard, given the clear independent influence of biophysical properties on thermoregulatory control, future studies should look to perform a more comprehensive examination of thermoregulatory-induced sweating during exercise in this vulnerable population, whereby all between-group differences in biophysical factors have been accounted for (i.e., H_prod_ and body mass and surface area).

## 4. Age-Related Changes in Skin Blood Flow

In addition to evaporative heat loss, the ability to adjust cutaneous vasomotor tone provides an effective means of managing a thermal load by redistributing cardiac output to modulate SkBF [45]. Indeed, this allows for the convective transfer of heat content within the circulatory system from the core to the periphery. These responses enable the distribution of internal heat content amongst various tissues in the body, as well as potentially raising skin temperature to facilitate increases in dry heat loss or minimise the rate of dry heat gain when the gradient for dry heat exchange is reversed (provided that the ambient temperature was greater than skin temperature) at a given ambient temperature. Therefore, the thermoregulatory-induced redistribution of blood flow to the skin is seen as a fundamental thermoregulatory response [46]. A common finding amongst studies that have examined thermoregulation in healthy aging to date is that older individuals demonstrate impaired heat-induced rises in SkBF compared to their younger counterparts [10, 11, 25, 26, 47–49]. Whilst aging does not appear to independently influence the onset threshold for cutaneous vasodilation (and accompanying changes in SkBF) [13], studies have consistently shown that older individuals exhibit attenuated increases in SkBF for a given change in core temperature, as well as lower time-dependant changes in SkBF compared to younger individuals [10, 11, 25, 26, 47–49]. This impaired cutaneous vascular response to heat stress reported in the aforementioned studies is observed even when older and younger individuals are matched for aerobic fitness, acclimation, and hydration status, suggesting that this is a primary function of human aging.

Whilst passive heat exposure stimulates increases in SkBF, it must be acknowledged that the combination of heat stress and exercise can further challenge the human cardiovascular system [50]. As such, the maintenance of blood pressure and skeletal muscle perfusion, in the face of increases in SkBF to meet thermoregulatory demands, necessitates an increase in cardiac output and redistribution of blood flow from inactive regions including the renal and splanchnic circulations [46]. In light of this, the combined independent demands from the skin, active skeletal muscle (both locomotor and respiratory), and blood pressure regulation will likely exceed available cardiac output [45]. In keeping with this suggestion, previous work has demonstrated that SkBF plateaus at ~60% of its maximum during exercise in the heat in healthy young individuals [51]. This is likely the result of a baroreceptor reflex-mediated control of SkBF imposed by exercise-induced metabolic demands to redistribute blood flow away from the cutaneous circulation to maintain optimal skeletal muscle and cerebral perfusion and blood pressure regulation [52]. If SkBF was to continue rising unabated during exercise, blood pressure would potentially fall which may then lead to a catastrophic perfusion failure of vascular beds. With such changes evident in apparently healthy young individuals, it may be argued that cardiovascular and thermal strain would be exacerbated to a greater extent in older individuals when exercising in a hot environment. The fact that SkBF responses during exercise in the heat are diminished to a much greater extent in older individuals compared to younger individuals, thereby negatively affecting heat content distribution and possibly even heat loss capacity in this population, lends some support to this suggestion [25].

Over the past several years, a number of researchers have examined the mechanisms underlying age-related impairments in cutaneous vasomotor control [9, 10, 13, 26, 38, 53–60]. From these investigations, it has been recognised that impairments in the mechanisms mediating cutaneous vasomotor control occur at multiple sites along the efferent arm of the sympathetic nervous system. These impairments include reductions in sympathetic cholinergic cotransmitter release, alterations in downstream signalling for vascular endothelial function, and attenuation in heat stress-induced sympathetic neural drive. Furthermore, it is important to note that age-related alterations in cardiovascular function and fluid status can also contribute to impaired SkBF responses during heat stress in older individuals. These age-related factors are discussed in greater detail below.

Thermoregulatory-induced rises in SkBF are primarily mediated by a sympathetic cholinergic active vasodilator system [61, 62]. Active cutaneous vasodilation is mediated through the release of acetylcholine and unknown cotransmitters, which facilitate cutaneous vasodilation through NO-dependant mechanisms [61, 62]. Studies have shown that older individuals exhibit an impaired vasodilatory response to hyperthermia and can be attributed to decreased sensitivity of the active vasodilator system [60, 63]. This decreased sensitivity of the active vasodilator system results in reduced cotransmitter signalling and, thus, attenuated NO-dependent cutaneous vasodilation. Hence, compared to younger individuals, older individuals predominately rely on compromised nitric oxide- (NO-) dependant cutaneous vasodilation to increase SkBF in response to environmental heat exposure and/or physical activity [54, 56, 57]. Furthermore, increases in skin and core body temperature elicit robust increases in skin sympathetic nerve activity in young healthy individuals [64]. However, when compared to young individuals, Stanhewicz et al. [58] not only demonstrated that older individuals exhibit less sympathetic outflow to the skin during passive heat stress, but also have an attenuated vasodilator response for a given increase in skin sympathetic nerve activity. These findings are consistent with that of Grassi et al. [53] who showed that skin sympathetic nerve activity modulation from mild changes in ambient temperature (±8°C) is markedly reduced in older individuals. Since increases in skin sympathetic nerve activity are thought to drive cutaneous vasodilator responses, overall these studies suggest that differences in sympathetic activity may contribute to the altered SkBF response observed in older individuals during heat stress.

Previous studies have also examined the cardiovascular response to heat stress. Findings from these studies have shown that capillary density in aged skin is reduced [65, 66] and that age-related reductions in SkBF are secondary to less cardiac output and blood flow redistribution from inactive visceral areas such as the renal and splanchnic circulations [26]. Minson et al. [26] demonstrated that smaller heat-induced rises in cardiac output were primarily due to a lower stroke volume, since the older individuals were able to increase their heart rate to a similar extent as the young individuals during passive heat stress. Similarly, Gagnon et al. [59] demonstrated that older individuals exhibit a lower cardiac output during passive heat stress compared to younger individuals. However, these authors reported that the Frank-Starling relation appropriately shifts during passive heat stress in older individuals, resulting in maintained stroke volume despite reductions in cardiac filling pressures and that the lower cardiac output observed was primarily mediated by a blunted heat-induced chronotropic response [59]. At present, the explanation for the differing findings between Minson et al. [26] and Gagnon et al. [59] is unclear; however, it may be that methodological differences between studies were responsible, as previously discussed [59].

In addition to the above, it has previously been proposed that less cardiac output and blood flow redistribution during heat stress may be due to age-related changes in fluid status [49, 67, 68]. Indeed, studies have shown that older individuals exhibit decrements in thirst sensation [69, 70], and renal sodium- and water-conserving capabilities decline with advancing age [71]. As such, these findings suggest that the capacity to accommodate large increases in intravascular blood volume and that the amount of blood available to circulate through the cutaneous vasculature are limited in older individuals compared to their younger counterparts. Taken together, this would imply that older individuals have an impaired ability to redistribute internal heat content amongst various tissues in the body and that internal heat storage is concentrated more toward the body core during heat stress.

## 5. Thermoregulation, Fitness, and Aging

Common practice for studies isolating the independent effect of a particular physiological factor (e.g., age) on thermoregulation during exercise is to match groups for peak oxygen uptake ($\dot{V}O_{2peak}$) and/or prescribe exercise using relative workloads [14, 25, 39, 40, 48, 72]. The rationale to use such a protocol is based on the original findings of Saltin and Hermansen [73], who demonstrated that exercise at the same %$\dot{V}O_{2peak}$ yielded similar core body temperature responses in young healthy individuals, irrespective of aerobic capacity. Later in 1989, Greenhaff [74] also reported smaller changes in core body temperature during exercise at a fixed absolute intensity in young fit ($\dot{V}O_{2peak}$: 60-65 ml/min/kg) compared to unfit ($\dot{V}O_{2peak}$: 40-45 ml/min/kg) individuals. Similar findings were further observed by Fritzche and Coyle [75] and Gant et al. [76] during exercise at a fixed %$\dot{V}O_{2peak}$ in a similar climate (~23°C). Collectively, these findings have led to the long-held notion that interindividual variability in core body temperature regulation during exercise is eliminated when the exercise intensity is expressed as a %$\dot{V}O_{2peak}$ [77].

Over the last 15 years, there has been a paradigmatic shift from the concept that core body temperature is driven according to %$\dot{V}O_{2peak}$, to an appreciation that the use of a fixed %$\dot{V}O_{2peak}$ protocol leads to different rates of H_prod_ between independent groups who are not matched for absolute $\dot{V}O_{2peak}$ [41, 42]. Accordingly, the participant group with the higher $\dot{V}O_{2peak}$ generates a greater level of H_prod_ during exercise at a fixed %$\dot{V}O_{2peak}$. As such, a greater rate of net heat loss is required to offset the greater rate of H_prod_ elicited by the experimental protocol [29]. Consistent with this suggestion is that greater sweat rates are commonly reported with increasing levels of fitness when exercise is prescribed at a fixed %$\dot{V}O_{2peak}$ [40, 78–80]. As such, previous studies that used a %$\dot{V}O_{2peak}$ approach might have incorrectly ascribed differences in thermoregulation to the physiological factor that was thought to be isolated/under investigation (e.g., age) [14, 25, 39, 40, 48, 72], when different rates of H_prod_ and, thus, requirements for heat loss may instead have been accountable.

In accordance with this theory, Jay et al. [42] examined the change in core body temperature during exercise at a fixed %$\dot{V}O_{2peak}$ and at a fixed absolute exercise intensity (and thus requirement for heat loss) in fit ($\dot{V}O_{2peak}$: ~60 ml/kg/min) and unfit ($\dot{V}O_{2peak}$: ~40 ml/kg/min) men matched for age (~22 y) and body mass (~78 kg). These authors demonstrated that the changes in core body temperature and sweat rates were greater in the fit group compared to the unfit group during exercise at 60%  $\dot{V}O_{2peak}$. In contrast, the changes in core body temperature and sweat rates were similar between groups during exercise at a fixed absolute exercise intensity (despite large differences in the relative exercise intensity). These findings support those of a classic study by Nielsen [81] who demonstrated that changes in core body temperature were determined by the absolute exercise intensity (and thus H_prod_), irrespective of aerobic fitness. Therefore, these findings suggest that the individual level of fitness may not influence one's ability to regulate changes in core body temperature during exercise.

It must be acknowledged that the findings of Jay et al. [42] can only be applicable to younger individuals who likely exhibit no physiological impairment in their ability to regulate changes core temperature. Given that there is evidence suggesting that older individuals exhibit impaired thermoregulation during exercise compared to their younger counterparts [14, 25, 39, 40, 48, 72], it must be noted that these studies used a %$\dot{V}O_{2peak}$ protocol. Therefore, it is difficult to determine whether the reported findings in these studies are due to aging* per se* [14, 25, 39, 40, 48, 72] or are simply ascribed to differences in the absolute H_prod_ and heat loss requirements, secondary to the use of a %$\dot{V}O_{2peak}$ protocol. In this regard, it is surprising that no study to date has used a similar experimental protocol as that described by Jay et al. [42] to elucidate the true independent influence of age and fitness on thermoregulatory responses during exercise. Hence, we believe that this is an area that requires further examination.

## 6. Clinical Implications of Age-Related Decrements in Thermoregulatory Control

In the preceding sections we have clearly identified that elderly individuals exhibit diminished sweating and SkBF responses during environment and/or physical activity-induced heat stress compared to their younger counterparts. As such, elderly individuals are likely limited in their ability to manage a thermal load secondary to a lower evaporative heat loss capacity and/or impairments in internal heat distribution. Based on the available evidence, we suspect that a lower sweat output per gland for a given level of cholinergic stimulation [35–37], as well as impaired intrinsic vasodilator pathways combined with a reduced cardiac reserve [25, 26, 65, 66], may contribute to this response in these individuals.

An important challenge for this field of research, and perhaps the most imperative, is to avoid older individuals from becoming discouraged from partaking in regular exercise [82]. Indeed, the health benefits associated with exercise still far outweigh the consequences of not performing exercise purely as a means to escape the potentially detrimental effects of hot weather [82]. Whilst current public health guidelines provide information on recommended levels of physical activity with associated long- and short-term beneficial outcomes, there are no recommendations regarding the levels of physical activity specifically for older individuals that can be safely performed in the heat (i.e., the summer months and bouts of hot weather) [15, 16]. Therefore, we believe there is urgent need for the development of public health recommendations for performing physical activity in the heat for older individuals, as well as future studies for developing strategies that enable older individuals' to optimally regulate their body temperature whilst performing routine physical activity.

In regard to strategies that may mitigate harmful elevations in heat strain, regular exercise training is known to improve several physiological parameters critical to thermoregulation. Indeed, exercise training has proven effective in improving cardiac [83, 84], autonomic [85], and vascular endothelial function and accompanying changes in blood flow distribution in older healthy individuals [86, 87]. As such, one may speculate that whilst exercise training is not only important for improving clinical health outcomes and preventing the onset of chronic disease [88], regular exercise training may also be important for maintaining and/or preventing the age-related decline in thermoregulatory control. In keeping with this suggestion are the findings of Inoue et al. [36] who demonstrated that regular exercise training slows the age-related decline in heat loss effector function, whereby sweat gland output in response to cholinergic stimulation is greater in fit older individuals compared to unfit older individuals. Moreover, plasma volume expansion with exercise training may allow for a larger heat-induced rise in cardiac output to facilitate a greater redistribution of blood flow to the skin and optimize heat content distribution amongst peripheral tissues [89]. Indeed, additional findings from Thomas et al. [90] and Okazaki et al. [48] showed that the core body temperature onset threshold for SkBF and sweating was improved in older men following both aerobic and resistance exercise training. From a thermoregulatory, these findings suggest that exercise training may improve thermoregulatory control, by augmenting heat-induced SkBF and sweating responses and thus potential internal heat distribution and evaporative heat loss capacity in older individuals.

Similar to regular exercise training, studies have reported that consecutive days (typically >6 days) of passive heat exposure (above 40°C for a minimum of 60 min) resulted in physiological adaptation to heat stress in older individuals [11, 36, 91, 92]. Physiological changes observed in these studies for older individuals included a lower core temperature onset threshold for sweating and SkBF, enhanced sweating and SkBF for a given absolute core body temperature, and reduced core body temperatures. In keeping with these findings, Best et al. [93] demonstrated that the reduction in resting core body temperature and increased SkBF and sweat production following 6 consecutive days of heat acclimation (60 min, 40% relative humidity) in older cyclists were similar to that observed in younger cyclists. These findings suggest that even older individuals who are habitually active can achieve similar physiological adaptations during consecutive bouts of heat stress as younger individuals. Whilst the above findings highlight the beneficial effect of acclimation on thermoregulatory control, Kohara et al. [94] recently reported that the age-related decline in vascular endothelial function, as evidenced by lower brachial artery pulse wave velocity, is attenuated in individuals who undertake a hot (>41°C) water bath compared to those who undertake a warm (<40°C) water bath more than 5 times per week (over four years). Moreover, a large cohort study (n = 2327) demonstrated that repeated whole-body heat exposure is associated with a marked reduction (~5 fold) in fatal cardiovascular and all-mortality events in men aged 42-60 years [95].

In addition to the above, it has previously been hypothesised that dietary supplements such as folic acid and nitrate (beetroot juice) and the use of electric fans might be beneficial in protecting against heat-related strain in those with thermoregulatory dysfunction [96]. Although theoretical at this time point, future studies are needed to examine the implications of these strategies directly in vulnerable populations. Indeed, this information would be invaluable given that climate change is predicted to bring about a greater variability in weather patterns with an increase in extreme weather events such as sustained heat waves [3, 97–99]. This, combined with the rising costs of electrical energy, which could make affordable electronic cooling devices (air-conditioning units) inaccessible to low-income individuals [3, 100, 101], could have a direct impact on population health since heat waves and/or bouts of hot weather can potentially exceed the physiological limit of compensability of vulnerable individuals, particularly the elderly.

## 7. Comorbid Disease and Thermoregulatory Control

Impaired thermoregulatory responses should be considered in older individuals as they may contribute to heat-related illness and, therefore, adversely impact upon health outcomes during everyday activities, including exercise, particularly during bouts of hot weather. Indeed, identifying potential thermoregulatory dysfunction during exercise in older individuals is important for a variety of reasons. There is now evidence that regular physical activity in this population increases exercise capacity and health-related quality of life and improves morbidity and mortality [15, 16]. However, with potentially suboptimal heat management capabilities, older individuals may become discouraged or even prevented, from participating in regular exercise, resulting in an increased risk of developing chronic heart and lung disease and hence a poorer health-related quality of life.

**Figure 2:**
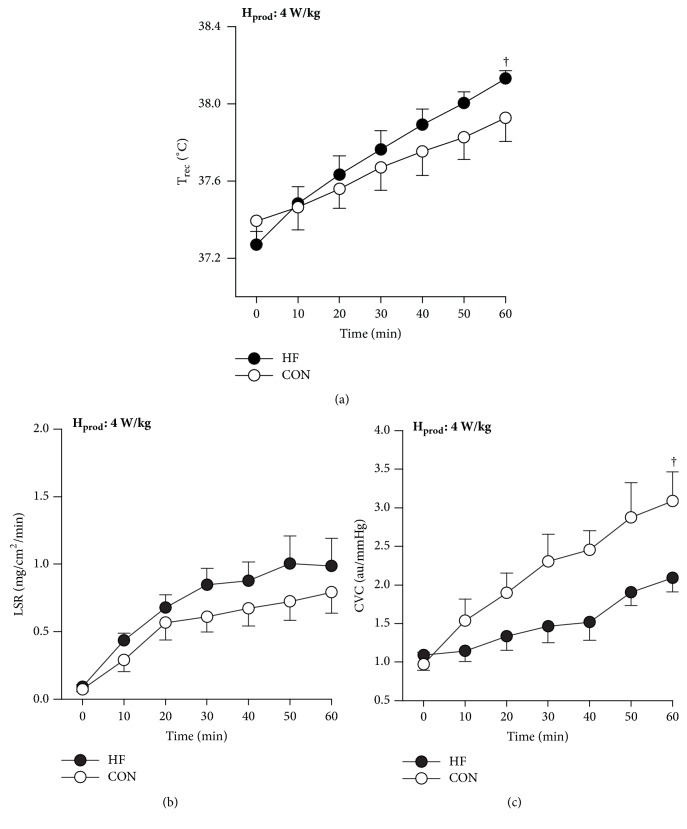
T_rec_ (a), CVC (b), and LSR (c) values recorded at 10-min intervals during the submaximal cycling test. H_prod_: metabolic heat production; T_rec_: rectal temperature; CVC: cutaneous vascular conductance; LSR: local sweat rate. Data are mean ± standard error of the mean. ^†^Significant group-time interaction, p < 0.05. Figure adapted from Balmain et al. [28].

Risks for heat-related illness are compounded for people with cardiovascular disease. Notably, we have recently demonstrated that thermoregulatory responses in individuals with heart failure differ from age-matched (~61 yr) healthy controls during exercise in a warm environment [28, 96, 102]. When exercising at the same rate of H_prod_ in a warm environment (30°C), we found that heart failure patients have a greater rise in core temperature compared to healthy controls (Figure 2, Panel (a)). Although sweating responses were preserved in heart failure (Figure 2, Panel (b)), cutaneous vascular conductance was greatly attenuated (due to a lower rise in SkBF) in these patients relative to healthy controls (Figure 2, Panel (c)). As such, patients with heart failure have a greater susceptibility to heat strain (greater rise in core body temperature) for a given combination of activity and climate compared to their age-matched healthy counterparts, secondary to poorer circulation to the periphery [96]. Therefore, our results suggest that thermoregulatory dysfunction and the associated risk of heat-related illness are compounded for people with heart failure.

It is important to note that studies examining thermoregulatory control in heart failure to date have included patients who continued with standard therapy [28, 51, 102–107]. Beta-blockade is a standard, first-line therapy for heart failure, which may possibly contribute to the lower rise in SkBF in these patients, particularly since younger individuals taking beta-blockers exhibit attenuated SkBF responses during exercise in the heat [108]. Moreover, diuretics may also influence thermoregulation. In healthy individuals, the use of diuretics has been associated with attenuated SkBF responses secondary to a reduction in plasma volume [109]. As such, patients with heart failure taking diuretics may impair thermoregulatory control during periods of heat stress. The fact that fluid status is also a fine balance in heart failure may predispose these patients to heat-related illness, should they become dehydrated, particularly during an exercise challenge [96].

Similar to individuals with heart failure, a number of clinical reports suggest that individuals with chronic lung disease, including asthma and chronic obstructive pulmonary disease, may be particularly vulnerable to heat-related illness during environmental heat exposure [110–113]. In addition, Jehn et al. [114] recently demonstrated that exposure to heat stress is associated with worse symptoms and a reduction in physical activity levels in individuals with pulmonary arterial hypertension. Whilst it appears that individuals with chronic lung disease are at a greater risk of heat-related morbidity and mortality, there is no evidence to date suggesting whether this is due to physiological impairments in the regulation of body temperature as a result of chronic conditions or simply can be ascribed to changes in air quality that typically accompany bouts of hot weather [115, 116]. Hence, we believe that thermoregulation in the context of chronic lung disease is an area that requires further examination.

## 8. Conclusion

Recent observations clearly show that older individuals are susceptible to heat-related illness during environmental and/or physical activity-induced heat stress. This increased susceptibility appears to be mediated by diminished heat-induced increases in sweating and SkBF responses. Based on this evidence, aging is clearly associated with an attenuated physiological ability to dissipate heat. It is also important to note that with an aging population and the projected consequences of global warming, a greater number of individuals, including those individuals with chronic heart and lung disease, will be exposed to the risks of heat-related morbidity and mortality in the coming decades. Hence, scope exists for further studies examining mechanisms of temperature regulation in the elderly to optimize the management of these vulnerable individuals when exposed to heat stress. Indeed, participation in physical activity is likely to be enhanced if environmental heat stress does not pose a significant barrier or safety concern for such individuals.

## References

[1] Patz J. A., Campbell-Lendrum D., Holloway T., Foley J. A. (2005). Impact of regional climate change on human health. *Nature*.

[2] Coates L., Haynes K., O'Brien J., McAneney J., De Oliveira F. D. (2014). Exploring 167 years of vulnerability: An examination of extreme heat events in Australia 1844-2010. *Environmental Science & Policy*.

[3] Patz J. A., Frumkin H., Holloway T., Vimont D. J., Haines A. (2014). Climate change: Challenges and opportunities for global health. *Journal of the American Medical Association*.

[4] Patz J. A., Khaliq M. (2002). Global climate change and health: Challenges for future practitioners. *Journal of the American Medical Association*.

[5] Watts N., Adger W. N., Agnolucci P. (2015). Health and climate change: policy responses to protect public health. *The Lancet*.

[6] Hausfater P., Megarbane B., Dautheville S. (2010). Prognostic factors in non-exertional heatstroke. *Intensive Care Medicine*.

[7] Shibasaki M., Okazaki K., Inoue Y. (2013). Aging and thermoregulation. *The Journal of Physical Fitness and Sports Medicine*.

[8] Van Someren E. J. W. (2007). Thermoregulation and aging. *American Journal of Physiology-Regulatory, Integrative and Comparative Physiology*.

[9] Holowatz L. A., Kenney W. L. (2010). Peripheral mechanisms of thermoregulatory control of skin blood flow in aged humans. *Journal of Applied Physiology*.

[10] Holowatz L. A., Thompson-Torgerson C., Kenney W. L. (2010). Aging and the control of human skin blood flow. *Frontiers in Bioscience*.

[11] Armstrong C. G., Kenney W. L. (1993). Effects of age and acclimation on responses to passive heat exposure. *Journal of Applied Physiology*.

[12] Sagawa S., Shiraki K., Yousef M. K., Miki K. (1988). Sweating and cardiovascular responses of aged men to heat exposure. *The Journals of Gerontology. Series A, Biological Sciences and Medical Sciences*.

[13] Kenny G. P., Yardley J., Brown C., Sigal R. J., Jay O. (2010). Heat stress in older individuals and patients with common chronic diseases. *Canadian Medical Association Journal*.

[14] Inbar O., Morris N., Epstein Y., Gass G. (2004). Comparison of thermoregulatory responses to exercise in dry heat among prepubertal boys, young adults and older males. *Experimental Physiology*.

[15] American College of Sports M, Chodzko-Zajko W. J., Proctor D. N. (2009). American College of Sports Medicine position stand. Exercise and physical activity for older adults. *Medicine and science in sports and exercise*.

[16] Sports Medicine Australia Hot weather guidelines for sporting clubs and associations and the physically active.

[17] Benzinger T. H. (1969). Heat regulation: homeostasis of central temperature in man.. *Physiological Reviews*.

[18] Kenny G. P., Jay O. (2013). Thermometry, calorimetry, and mean body temperature during heat stress. *Comprehensive Physiology*.

[19] Parsons K. (2014). *Human Thermal Environments: The Effects of Hot, Moderate, and Cold Environments on Human Health, Comfort, and Performance*.

[20] Gleeson M. (1998). Temperature regulation during exercise. *International Journal of Sports Medicine*.

[21] Whipp B. J., Wasserman K. (1969). Efficiency of muscular work.. *Journal of Applied Physiology*.

[22] Boulant J. A. (2000). Role of the preoptic-anterior hypothalamus in thermoregulation and fever. *Clinical Infectious Diseases*.

[23] Epstein Y., Roberts W. O. (2011). The pathopysiology of heat stroke: an integrative view of the final common pathway. *Scandinavian Journal of Medicine & Science in Sports*.

[24] Leon L. R., Bouchama A. (2015). Heat stroke. *Comprehensive Physiology*.

[25] Kenney W. L., Morgan A. L., Farquhar W. B., Brooks E. M., Pierzga J. M., Derr J. A. (1997). Decreased active vasodilator sensitivity in aged skin. *American Journal of Physiology-Heart and Circulatory Physiology*.

[26] Minson C. T., Wladkowski S. L., Cardell A. F., Pawelczyk J. A., Kenney W. L. (1998). Age alters the cardiovascular response to direct passive heating. *Journal of Applied Physiology*.

[27] Inoue Y. (1996). Regional differences in age related decrements of the cutaneous vascular and sweating responses to passive heating. *European Journal of Applied Physiology*.

[28] Balmain B. N., Jay O., Morris N. R. (2018). Thermoeffector Responses at a Fixed Rate of Heat Production in Heart Failure Patients. *Medicine and science in sports and exercise*.

[29] Gagnon D., Jay O., Kenny G. P. (2013). The evaporative requirement for heat balance determines whole-body sweat rate during exercise under conditions permitting full evaporation. *The Journal of Physiology*.

[30] Lind A. R. (1963). A physiological criterion for setting thermal environmental limits for everyday work. *Journal of Applied Physiology*.

[31] Wenger C. B. (1972). Heat of evaporation of sweat: thermodynamic considerations.. *Journal of Applied Physiology*.

[32] Dufour A., Candas V. (2007). Ageing and thermal responses during passive heat exposure: Sweating and sensory aspects. *European Journal of Applied Physiology*.

[33] Inoue Y., Kuwahara T., Araki T. (2004). Maturation- and Aging-related Changes in Heat Loss Effector Function. *Journal of Physiological Anthropology and Applied Human Science*.

[34] Inoue Y., Nakao M., Okudaira S., Ueda H., Araki T. (1995). Seasonal variation in sweating responses of older and younger men. *European Journal of Applied Physiology*.

[35] Kenney W. L., Fowler S. R. (1988). Methylcholine-activated eccrine sweat gland density and output as a function of age. *Journal of Applied Physiology*.

[36] Inoue Y., Havenith G., Kenney W. L., Loomis J. L., Buskirk E. R. (1999). Exercise- and methylcholine-induced sweating responses in older and younger men: Effect of heat acclimation and aerobic fitness. *International Journal of Biometerology*.

[37] Inoue Y., Shibasaki M., Ueda H., Ishizashi H. (1999). Mechanisms underlying the age-related decrement in the human sweating response. *European Journal of Applied Physiology*.

[38] Kenney W. L., Munce T. A. (2003). Aging and human temperature regulation. *Journal of Applied Physiology*.

[39] Kenney W. L., Anderson R. K. (1988). Responses of older and younger women to exercise in dry and humid heat without fluid replacement. *Medicine & Science in Sports & Exercise*.

[40] Tankersley C. G., Smolander J., Kenney W. L., Fortney S. M. (1991). Sweating and skin blood flow during exercise: Effects of age and maximal oxygen uptake. *Journal of Applied Physiology*.

[41] Cramer M. N., Jay O. (2014). Selecting the correct exercise intensity for unbiased comparisons of thermoregulatory responses between groups of different mass and surface area. *Journal of Applied Physiology*.

[42] Jay O., Bain A. R., Deren T. M., Sacheli M., Cramer M. N. (2011). Large differences in peak oxygen uptake do not independently alter changes in core temperature and sweating during exercise. *American Journal of Physiology-Regulatory, Integrative and Comparative Physiology*.

[43] Gagge A. P., Gonazalez R. R. (2011). Mechanisms of heat exchange: biophysics and physiology. *Comprehensive Physiology*.

[44] Cramer M. N., Jay O. (2016). Biophysical aspects of human thermoregulation during heat stress. *Autonomic Neuroscience: Basic and Clinical*.

[45] Rowell L. B. (1974). Human cardiovascular adjustments to exercise and thermal stress. *Physiological Reviews*.

[46] Johnson J. M. (2010). Exercise in a hot environment: the skin circulation. *Scandinavian journal of medicine & science in sports*.

[47] Inoue Y., Shibasaki M., Hirata K., Araki T. (1998). Relationship between skin blood flow and sweating rate, and age related regional differences. *European Journal of Applied Physiology*.

[48] Okazaki K., Kamijo Y.-I., Takeno Y., Okumoto T., Masuki S., Nose H. (2002). Effects of exercise training on thermoregulatory responses and blood volume in older men. *Journal of Applied Physiology*.

[49] Kenney W. L., Tankersley C. G., Newswanger D. L., Hyde D. E., Puhl S. M., Turner N. L. (1990). Age and hypohydration independently influence the peripheral vascular response to heat stress. *Journal of Applied Physiology*.

[50] González-alonso J., Crandall C. G., Johnson J. M. (2008). The cardiovascular challenge of exercising in the heat. *The Journal of Physiology*.

[51] Zelis R., Mason D. T., Braunwald E. (1969). Partition of blood flow to the cutaneous and muscular beds of the forearm at rest and during leg exercise in normal subjects and in patients with heart failure.. *Circulation Research*.

[52] Kellogg D. L., Johnson J. M., Kenney W. L., Pergola P. E., Kosiba W. A. (1993). Mechanisms of control of skin blood flow during prolonged exercise in humans. *American Journal of Physiology-Heart and Circulatory Physiology*.

[53] Grassi G., Seravalle G., Turri C., Bertinieri G., Dell'Oro R., Mancia G. (2003). Impairment of thermoregulatory control of skin sympathetic nerve traffic in the elderly. *Circulation*.

[54] Holowatz L. A., Houghton B. L., Wong B. J. (2003). Nitric oxide and attenuated reflex cutaneous vasodilation in aged skin. *American Journal of Physiology-Heart and Circulatory Physiology*.

[55] Holowatz L. A., Jennings J. D., Lang J. A., Larry Kenney W. (2009). Ketorolac alters blood flow during normothermia but not during hyperthermia in middle-aged human skin. *Journal of Applied Physiology*.

[56] Minson C. T., Holowatz L. A., Wong B. J., Kenney W. L., Wilkins B. W. (2002). Decreased nitric oxide- and axon reflex-mediated cutaneous vasodilation with age during local heating. *Journal of Applied Physiology*.

[57] Reckelhoff J. F., Kellum J. A., Blanchard E. J., Bacon E. E., Wesley A. J., Kruckeberg W. C. (1994). Changes in nitric oxide precursor, L-arginine, and metabolites, nitrate and nitrite, with aging. *Life Sciences*.

[58] Stanhewicz A. E., Greaney J. L., Alexander L. M., Kenney W. L. (2016). Blunted increases in skin sympathetic nerve activity are related to attenuated reflex vasodilation in aged human skin. *Journal of Applied Physiology*.

[59] Gagnon D., Romero S. A., Ngo H. (2016). Healthy aging does not compromise the augmentation of cardiac function during heat stress. *Journal of Applied Physiology*.

[60] Charkoudian N. (2003). Skin blood flow in adult human thermoregulation: how it works, when it does not, and why. *Mayo Clinic Proceedings*.

[61] Kellogg D. L. (2006). In vivo mechanisms of cutaneous vasodilation and vasoconstriction in humans during thermoregulatory challenges. *Journal of Applied Physiology*.

[62] Charkoudian N. (2010). Mechanisms and modifiers of reflex induced cutaneous vasodilation and vasoconstriction in humans. *Journal of Applied Physiology*.

[63] Kenney W. L., Morgan A. L., Farquhar W. B. (1997). Decreased active vasodilator sensitivity in aged skin. *American Journal of Physiology-Heart and Circulatory Physiology*.

[64] Cui J., Sathishkumar M., Wilson T. E., Shibasaki M., Davis S. L., Crandall C. G. (2006). Spectral characteristics of skin sympathetic nerve activity in heat-stressed humans. *American Journal of Physiology-Heart and Circulatory Physiology*.

[65] Fenske N. A., Lober C. W. (1986). Structural and functional changes of normal aging skin. *Journal of the American Academy of Dermatology*.

[66] Smith L. (1989). Histopathologic characteristics and ultrastructure of aging skin. *Cutis; Cutaneous Medicine for the Practitioner*.

[67] Nadel E. R., Fortney S. M., Wenger C. B. (1980). Effect of hydration state of circulatory and thermal regulations. *Journal of Applied Physiology*.

[68] Sawka M. N., Montain S. J., Latzka W. A. (2001). Hydration effects on thermoregulation and performance in the heat. *Comparative Biochemistry and Physiology - A Molecular and Integrative Physiology*.

[69] Rolls B. J., Phillips P. A. (1990). Aging and Disturbances of Thirst and Fluid Balance. *Nutrition Reviews*.

[70] Kenney W. L., Chiu P. (2001). Influence of age on thirst and fluid intake. *Medicine & Science in Sports & Exercise*.

[71] Mack G. W., Weseman C. A., Langhans G. W., Scherzer H., Gillen C. M., Nadel E. R. (1994). Body fluid balance in dehydrated healthy older men: Thirst and renal osmoregulation. *Journal of Applied Physiology*.

[72] Ho C. W., Beard J. L., Farrell P. A., Minson C. T., Kenney W. L. (1997). Age, fitness, and regional blood flow during exercise in the heat. *Journal of Applied Physiology*.

[73] Saltin B., Hermansen L. (1966). Esophageal, rectal, and muscle temperature during exercise.. *Journal of Applied Physiology*.

[74] Greenhaff P. L. (1989). Cardiovascular fitness and thermoregulation during prolonged exercise in man. *British Journal of Sports Medicine*.

[75] Fritzsche R. G., Coyle E. F. (2000). Cutaneous blood flow during exercise is higher in endurance-trained humans. *Journal of Applied Physiology*.

[76] Gant N., Williams C., King J., Hodge B. J. (2004). Thermoregulatory responses to exercise: Relative versus absolute intensity. *Journal of Sports Sciences*.

[77] Mora-Rodriguez R. (2012). Influence of aerobic fitness on thermoregulation during exercise in the heat. *Exercise and Sport Sciences Reviews*.

[78] Ichinose-Kuwahara T., Inoue Y., Iseki Y., Hara S., Ogura Y., Kondo N. (2010). Sex differences in the effects of physical training on sweat gland responses during a graded exercise. *Experimental Physiology*.

[79] Ichinose T. K., Inoue Y., Hirata M., Shamsuddin A. K. M., Kondo N. (2009). Enhanced heat loss responses induced by short-term endurance training in exercising women. *Experimental Physiology*.

[80] Mora-Rodriguez R., Coso J. D., Hamouti N., Estevez E., Ortega J. F. (2010). Aerobically trained individuals have greater increases in rectal temperature than untrained ones during exercise in the heat at similar relative intensities. *European Journal of Applied Physiology*.

[81] Nielsen M. (1938). The regulation of body temperature in Muskelarbeit1. *Scandinavian Archives of Physiology*.

[82] Waldock K., Hayes M., Watt P., Maxwell N. (2018). Physiological and perceptual responses in the elderly to simulated daily living activities in UK summer climatic conditions. *Public Health*.

[83] Spina R. J., Turner M. J., Ehsani A. A. (1998). *β*-Adrenergic-mediated improvement in left ventricular function by exercise training in older men. *American Journal of Physiology-Heart and Circulatory Physiology*.

[84] Ehsani A. A., Ogawa T., Miller T. R., Spina R. J., Jilka S. M. (1991). Exercise training improves left ventricular systolic function in older men. *Circulation*.

[85] Ueno L. M., Moritani T. (2003). Effects of long-term exercise training on cardiac autonomic nervous activities and baroreflex sensitivity. *European Journal of Applied Physiology*.

[86] Pierce G. L., Eskurza I., Walker A. E., Fay T. N., Seals D. R. (2011). Sex-specific effects of habitual aerobic exercise on brachial artery flow-mediated dilation in middle-aged and older adults. *Clinical Science*.

[87] Franzoni F., Ghiadoni L., Galetta F. (2005). Physical activity, plasma antioxidant capacity, and endothelium-dependent vasodilation in young and older men. *American Journal of Hypertension*.

[88] Warburton D. E. R., Nicol C. W., Bredin S. S. D. (2006). Health benefits of physical activity: the evidence. *Canadian Medical Association Journal*.

[89] Convertino V. A. (1991). Blood volume: Its adaptation to endurance training. *Medicine & Science in Sports & Exercise*.

[90] Thomas C. M., Pierzga J. M., Kenney W. L. (1999). Aerobic training and cutaneous vasodilation in young and older men. *Journal of Applied Physiology*.

[91] Aoyagi Y., McLellan T. M., Shephard R. J. (1997). Interactions of physical training and heat acclimation. The thermophysiology of exercising in a hot climate. *Sports Medicine*.

[92] Candas V., Libert J. P., Vogt J. J. (1979). Influence of air velocity and heat acclimation on human skin wettedness and sweating efficiency. *Journal of Applied Physiology: Respiratory, Environmental and Exercise Physiology*.

[93] Best S., Thompson M., Caillaud C., Holvik L., Fatseas G., Tammam A. (2014). Exercise-heat acclimation in young and older trained cyclists. *Journal of Science and Medicine in Sport*.

[94] Kohara K., Tabara Y., Ochi M. (2018). Habitual hot water bathing protects cardiovascular function in middle-aged to elderly Japanese subjects. *Scientific Reports*.

[95] Laukkanen T., Khan H., Zaccardi F., Laukkanen J. A. (2015). Association between sauna bathing and fatal cardiovascular and all-cause mortality events. *JAMA Internal Medicine*.

[96] Balmain B. N., Sabapathy S., Jay O. (2017). Heart Failure and Thermoregulatory Control: Can Patients With Heart Failure Handle the Heat?. *Journal of Cardiac Failure*.

[97] Ebi K. L., Exuzides K. A., Lau E., Kelsh M., Barnston A. (2004). Weather changes associated with hospitalizations for cardiovascular diseases and stroke in California, 1983–1998. *International Journal of Biometerology*.

[98] Perkins S. E., Alexander L. V., Nairn J. R. (2012). Increasing frequency, intensity and duration of observed global heatwaves and warm spells. *Geophysical Research Letters*.

[99] Portier C. J., Thigpen Tart K. T., Carter S. R. (2013). A Human Health Perspective on Climate Change: A Report Outlining Research Needs on the Human Health Effects of Climate Change. *Journal of Current Issues in Globalization*.

[100] Hajat S., O'Connor M., Kosatsky T. (2010). Health effects of hot weather: from awareness of risk factors to effective health protection. *The Lancet*.

[101] Lundgren K., Kjellstrom T. (2013). Sustainability challenges from climate change and air conditioning use in urban areas. *Sustainability*.

[102] Balmain B. N., Jay O., Sabapathy S. (2016). Altered thermoregulatory responses in heart failure patients exercising in the heat. *Physiological Reports*.

[103] Benda N. M. M., Eijsvogels T. M. H., Van Dijk A. P. J., Bellersen L., Thijssen D. H. J., Hopman M. T. E. (2016). Altered core and skin temperature responses to endurance exercise in heart failure patients and healthy controls. *European Journal of Preventive Cardiology*.

[104] Cui J., Arbab-Zadeh A., Prasad A., Durand S., Levine B. D., Crandall C. G. (2005). Effects of heat stress on thermoregulatory responses in congestive heart failure patients. *Circulation*.

[105] Cui J., Boehmer J. P., Blaha C., Lucking R., Kunselman A. R., Sinoway L. I. (2013). Chronic heart failure does not attenuate the total activity of sympathetic outflow to skin during whole-body heating. *Circulation: Heart Failure*.

[106] Green D. J., Maiorana A. J., Siong J. H. J. (2006). Impaired skin blood flow response to environmental heating in chronic heart failure. *European Heart Journal*.

[107] Morgan C. L., Nadas A. S. (1963). Sweating and Congestive Heart Failure. *The New England Journal of Medicine*.

[108] Freund B. J., Joyner M. J., Jilka S. M. (1987). Thermoregulation during prolonged exercise in heat: alterations with beta-adrenergic blockade. *Journal of Applied Physiology*.

[109] Fortney S. M., Nadel E. R., Wenger C. B., Bove J. R. (1981). Effect of blood volume on sweating rate and body fluids in exercising humans. *Journal of Applied Physiology*.

[110] Semenza J. C., McCullough J. E., Flanders W. D., McGeehin M. A., Lumpkin J. R. (1999). Excess hospital admissions during the July 1995 heat wave in Chicago. *American Journal of Preventive Medicine*.

[111] Semenza J. C., Rubin C. H., Falter K. H. (1996). Heat-related deaths during the July 1995 heat wave in Chicago. *The New England Journal of Medicine*.

[112] Schwartz J. (2005). Who is Sensitive to Extremes of Temperature?. *Epidemiology*.

[113] Hoffmann B., Hertel S., Boes T., Weiland D., Jöckel K.-H. (2008). Increased cause-specific mortality associated with 2003 heat wave in Essen, Germany. *Journal of Toxicology and Environmental Health, Part A. Current Issues*.

[114] Jehn M., Gebhardt A., Liebers U. (2014). Heat stress is associated with reduced health status in pulmonary arterial hypertension: A prospective study cohort. *Lung*.

[115] Papanastasiou D. K., Melas D., Kambezidis H. D. (2015). Air quality and thermal comfort levels under extreme hot weather. *Atmospheric Research*.

[116] Fischer P. H., Brunekreef B., Lebret E. (2004). Air pollution related deaths during the 2003 heat wave in the Netherlands. *Atmospheric Environment*.

